# Full-length genome sequences of Estonian HIV-1 CRF06 and Eastern-European subtype A1 recombinant forms

**DOI:** 10.1186/1471-2334-14-S4-O24

**Published:** 2014-05-29

**Authors:** Radko Avi, Kristi Huik, Merit Pauskar, Eveli Kallas, Ene-Ly Jõgeda, Kai Zilmer, Valentina Ustina, Lilia Novikova, Tõnis Karki, Veljo Kisand, Irja Lutsar

**Affiliations:** 1 Department of Microbiology, University of Tartu, Tartu, Estonia

## 

“Eastern-European type” of HIV-1 epidemic started among Estonian intravenous drug users (IDU) population in 2000. Unlike in other former Soviet Union countries the Estonian HIV-1 epidemic was not caused by subtype A1 viruses but recombinant form CRF06_cpx and its next generation recombinants with subtype A1. A high variety of different recombinant forms has also been described in other IDU driven HIV-1 epidemics in other countries.

Aim: to sequence and describe the subtypic structure of near-full genome Estonian HIV-1 inter-subtype recombinant forms in Estonian IDU population.

The study included plasma samples from 10 HIV positive subjects collected in 2000-2010. Samples were selected based on discordant subtypes or recombinant sequences in different genomic regions in recent Estonian studies. Patients’ viral genomic RNA was reverse transcribed, amplified, cloned and Sanger sequenced in four overlapping genomic regions. Phylogenetic trees were constructed using the Maximum-Likelihood method and recombination pattern was assessed using Simplot software (similarity and bootscanning blots). Drug resistance mutations and tropism were determined by Stanford HIV Drug Resistance Database and geno2pheno analysis tool using a false positive rate of 10%.

According to bootscanning analysis the majority of strains (80%) indicated unique recombination structure between Estonian CRF06_cpx and Russian subtypes A1 sequences, whereas prevailing proportions of regions belong to the CRF06_cpx clade. Phylogenetic tree analysis confirmed these results indicating monophyletic clustering with Estonian CRF06_cpx or Russian subtype A1 sequences in corresponding regions. Of 10 sequences in one NNRTI DRM K103N and V179E was found and in 9 INI accessory mutations L74I was found. Of seven gag region sequences in three cases protease inhibitor associated substitution L449F, and in one case V128I, P452S and maturation inhibitor substitutions V370M were present. Of 9 env sequences one strain was R4 tropic, the others were R5 tropic.

Analyzed sequences revealed high recombinational diversity of Estonian inter-subtype HIV-1 recombinant strains, which is also characteristic to other IDU HIV-1 epidemics. Nearly full-length genome sequences generated by this study provide reference material for high-throughput sequencing and for tracking the molecular epidemics in different risk groups and in Estonia and neighboring countries.

